# Circulating microRNAs Associated with Insulin Resistance in South Asian and European Adults with Type 2 Diabetes (T2D): A Systematic Review and Mapping of Ethnic Patterns

**DOI:** 10.3390/ijms27219827

**Published:** 2026-11-02

**Authors:** Talia Kimber, David J. Hunter, Elizabeth C. Akam, Monica Singh, Puneetpal Singh, Sarabjit S. Mastana

**Affiliations:** 1 School of Sport, Exercise and Health Sciences, Loughborough University, Loughborough LE11 3TU, UK; taliakimber@yahoo.com (T.K.); d.j.hunter@lboro.ac.uk (D.J.H.); e.c.akam@lboro.ac.uk (E.C.A.); 2 Department of Human Genetics, Punjabi University, Patiala 147002, India; singhmonica2017@gmail.com (M.S.); puneetpalsingh@pbi.ac.in (P.S.)

**Keywords:** type 2 diabetes, microRNAs, European, South Asian, insulin resistance, systematic review

## Abstract

Type 2 diabetes (T2D) is a global crisis affecting 589 million adults, with South Asian populations particularly vulnerable, developing T2D earlier and at lower BMIs than Europeans. Insulin resistance, measured by HOMA-IR, is central to T2D pathogenesis. Circulating microRNAs are stable biomarkers implicated in insulin signalling, but no systematic review has compared their associations with insulin resistance across South Asians and Europeans. To identify microRNAs associated with HOMA-IR in adults with T2D and explore whether association patterns differ between South Asians and Europeans. A PRISMA-compliant systematic review searched six databases (PubMed, Embase, Web of Science, Scopus, bioRxiv, medRxiv). Eligible studies reported statistical associations between circulating serum/plasma microRNA and HOMA-IR in adults with T2D. Bias risks were assessed using an adapted domain-based tool. A qualitative synthesis was conducted because of the differences in methodology. The review was registered on the Open Science Framework (OSF). A total of 14 studies were included (9 European, 5 South Asian), reporting 29 associations across 21 microRNA species, with 12 reaching statistical significance (*p* < 0.05). The strongest finding was that miR-122 positively associated with log HOMA-IR in a large European prospective cohort. Co-directional associations for miR192 and miR-194 suggested a hepatic microRNA cluster in Europeans. The miR-29 family and miR-192 emerged as cross-ethnic signals. Associations reported in South Asian populations included miR-128 and miR-374a-5p. Circulating microRNAs showed consistent positive associations with HOMA-IR, supporting biomarker potential. Cross-ethnic comparison was not possible within the included studies, revealing a gap in the literature rather than true ethnic differences. Multi-ethnic, methodologically standardised studies are needed, prioritising miR-122, miR-192, the miR-29 family, miR-128, and miR-374a-5p.

## 1. Introduction

Type 2 diabetes mellitus (T2D) is a chronic, multifactorial disorder, characterised by insulin resistance and insulin deficiency, resulting in persistent hyperglycaemia and progressive metabolic dysfunction [1,2,3]. T2D accounts for approximately 90–95% of all diabetes cases in developed countries, with a greater proportion in developing regions, making it the predominant form of diabetes worldwide [1,2]. T2D has become a major global public health concern. Population ageing, urbanisation, dietary changes and reduced physical activity [1,4,5], accompanied by rising levels of obesity and metabolic disorders, have contributed to the increasing prevalence of diabetes worldwide [3,4]. T2D contributes substantially to global mortality, with an estimated 3.4 million deaths in 2024, making it one of the leading causes of death worldwide [5,6].

T2D prevalence is projected to increase from 589 million in 2024 to 853 million by 2050, representing a 45% rise globally [6]. The largest increases are expected in middle-income countries, including much of South Asia [6], where the prevalence of diabetes has nearly doubled, rising from approximately 11.3% in 2000–2004 to 22.3% in 2020–2024 [6,7,8,9]. This trend has been linked to fast urbanisation, adoption of sedentary and calorie-dense Western dietary patterns, declining physical activity, and genetic susceptibilities unique to South Asian populations [9,10].

South Asians are disproportionately affected by T2D, developing the disease at younger ages and at lower body mass index (BMI) levels than Europeans [7,10,11]. This elevated risk has been observed both within South Asia and among migrant populations, suggesting contributions from both biological and environmental factors [8]. Increased insulin resistance, greater visceral adiposity, and central fat accumulation are thought to contribute to this susceptibility [7,10,11,12,13,14,15,16,17]. More broadly, T2D risk is influenced by complex interactions among genetic predisposition, early-life nutritional exposures, and metabolic pathways regulating insulin sensitivity [13,14,15,16,17,18,19,20].

Recent advances in genomics and molecular biology have provided new insights into T2D pathogenesis. Genome-wide association studies have identified numerous susceptibility loci, with increasing attention paid to epigenomic and post-transcriptional regulatory mechanisms as important contributors to disease pathogenesis [18,21]. Among these, miRNAs have emerged as particularly promising molecular regulators, with evidence implicating them in the regulation of insulin signalling, glucose metabolism, and pancreatic β-cell function [21,22].

### MicroRNAs: Biology and Role in Type 2 Diabetes

**Figure 1:**
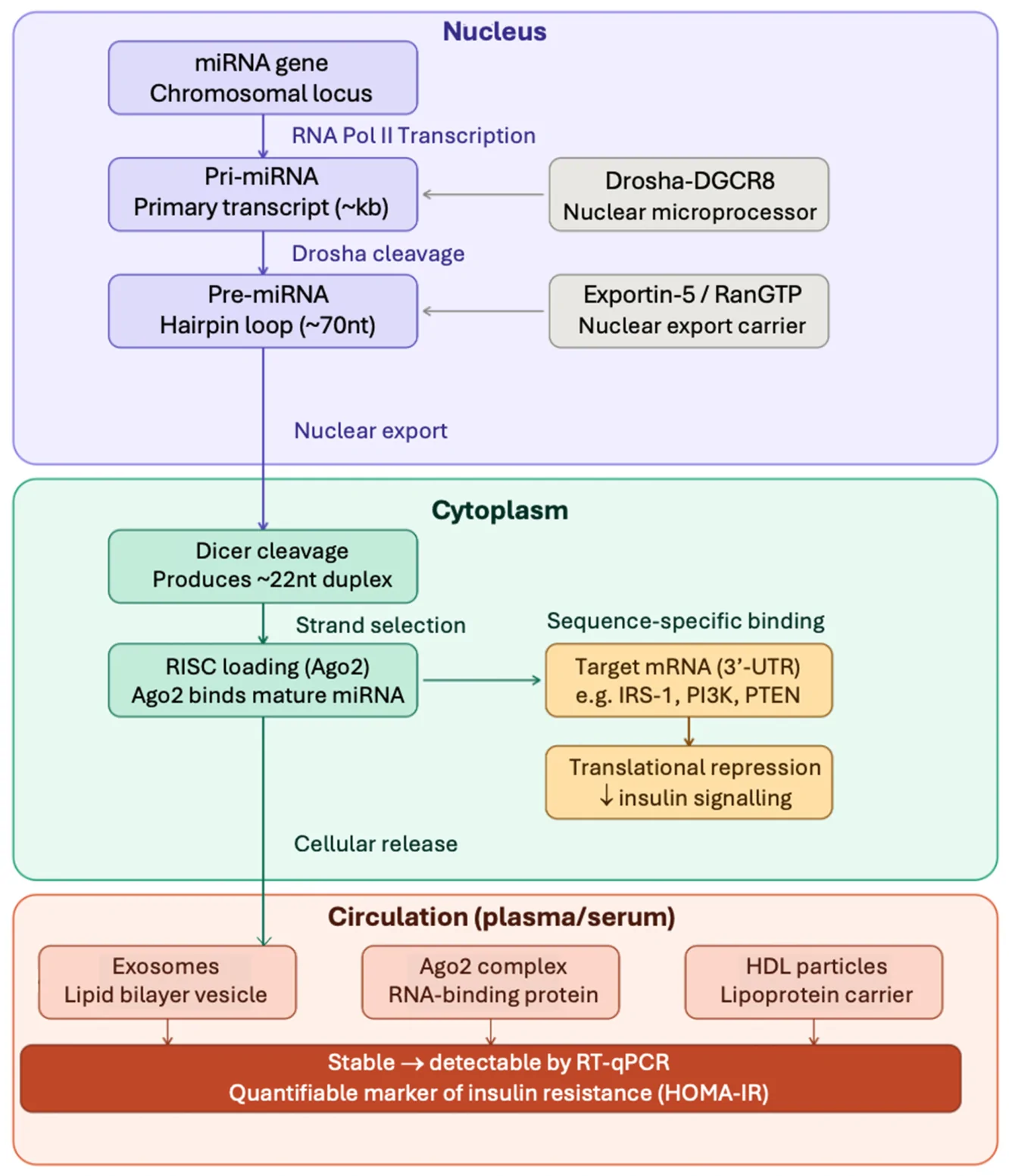
Schematic overview of miRNA biogenesis, cellular release, and target gene silencing. In the nucleus, miRNA genes are transcribed and processed by Drosha-DGCR8 to yield pre-miRNA hairpins, which are exported via Exportin-5/RanGTP. In the cytoplasm, Dicer cleaves the hairpin into a mature ~22 nt duplex loaded into RISC (Ago2), enabling translational repression of target mRNAs including insulin signalling components (IRS-1, PI3K, PTEN). Mature miRNAs are released into circulation encapsulated in exosomes, bound to Ago2, or associated with HDL particles, providing stability and enabling detection by RT-qPCR as biomarkers of insulin resistance. Deng and Guo 2019 [22]; Turchinovich et al., 2012 [23]; Valadi et al., 2007 [24]; Mitchell et al., 2008 [25].

MicroRNAs (miRNAs) are small non-coding RNAs, around 22 nucleotides long, that regulate gene expression post-transcriptionally by binding to complementary sequences within the 3′-UTR of mRNAs and commonly promoting translational repression or degradation of the target transcript. miRNAs are critical in controlling numerous processes, including cellular differentiation, proliferation, metabolism and homeostasis [22]. A single miRNA can regulate multiple target genes, allowing these molecules to act as important modulators of complex biological pathways [22,23,24,25]. The key steps relevant to miRNAs and insulin signalling are illustrated in Figure 1.

Dysregulation of specific miRNAs can influence key metabolic pathways involved in insulin signalling, glucose homeostasis and pancreatic β-cell function [26,27]. Several miRNAs regulate components of the insulin signalling cascade, including molecules within the PI3K–AKT (Phosphoinositide 3-kinase-Protein Kinase B) pathway, which is central to insulin-mediated glucose uptake in metabolic tissues [22,26]. Other miRNAs influence pancreatic β-cell development and insulin secretion, highlighting their involvement in multiple stages of diabetes pathogenesis [26,27].

Circulating miRNAs are detectable in blood and remain sufficiently stable for biomarker research as they are protected from degradation through association with extracellular vesicles, RNA-binding proteins, and lipoproteins [23,24]. This stability, together with the minimally invasive nature of blood sampling, has made circulating serum and plasma miRNAs preferential for biomarker studies [23,24,25].

In metabolic disease, circulating miRNAs have been reported to differ between individuals with T2D or insulin resistance compared to healthy controls. For example, miR-21-5p, miR-34a-5p, miR-146a-5p and miR-126-3p have been consistently identified as dysregulated across multiple studies [26,27,28]. Altered levels of circulating miRNAs have also been shown to correlate with markers of insulin resistance, suggesting their potential utility as biomarkers that reflect underlying pathophysiological changes relevant to T2D [26,27,28]. Studies investigating circulating miRNAs and insulin resistance have examined many candidate molecules, often within small cohorts. As a result, there is limited understanding of whether patterns of circulating miRNA dysregulation are consistent across different populations. This represents a significant gap in the literature, particularly given the substantial ethnic differences observed in the prevalence and metabolic characteristics of T2D.

South Asians are particularly underrepresented in medical and genomic studies. Despite comprising approximately 25% of the world’s population, South Asians account for less than 2% of participants in genomic studies [29], with similarly limited representation in miRNA research. Importantly, ethnic differences in circulating miRNA profiles may not simply reflect epidemiological or phenotypic disparities but may also have a direct molecular basis. Population-specific genetic variation can influence miRNA sequence, expression, and target binding [30], while ancestry-related differences in miRNA eQTLs suggest that the same circulating miRNA may show different basal expression across ethnic groups [31,32].

No systematic review has compared circulating miRNA signatures associated with insulin resistance between South Asian and European populations. A scoping search of PROSPERO and Cochrane found no prior/in-progress review on this topic. This systematic review aims to identify circulating miRNAs associated with insulin resistance, measured using HOMA-IR, in adults with T2D and to explore whether association patterns differ across South Asian and European studies. A secondary aim is to map the existing cross-ethnic evidence base and identify structural gaps that current biomarker research programmes may reinforce to facilitate T2D diagnosis and therapeutic interventions in different ethnic populations.

## 2. Materials and Methods

### 2.1. Study Design

This systematic review followed PRISMA 2020 guidelines [33] and identified, critically appraised, and synthesised evidence from observational studies investigating associations between circulating miRNA expression and insulin resistance, quantified by HOMA-IR, in South Asian and European adults with T2D. Meta-analysis was not appropriate due to substantial clinical and methodological heterogeneity in miRNAs measured, laboratory methods, HOMA-IR handling, adjustment strategies, and biological sample types, making any pooled estimate difficult to interpret, with limited biological or statistical value. A qualitative synthesis was therefore considered the most appropriate and is consistent with the Synthesis Without Meta-analysis (SWiM) guidance for reviews [34]. The review was registered on the Open Science Framework (OSF) (https://osf.io/zkqyj/overview, accessed on 9 April 2026).

### 2.2. Eligibility Criteria

Eligibility criteria were pre-specified prior to database searching and applied consistently at both the title/abstract and full-text screening stages. Criteria were structured according to the PICOS framework (Population, Intervention/Exposure, Comparator, Outcome, Study design) and entered into Covidence [35]. The full inclusion and exclusion criteria are presented in Table 1.

### 2.3. Search Strategy

Six electronic databases were searched to identify eligible studies: PubMed, Embase, Web of Science, Scopus, bioRxiv, and medRxiv. The preprint servers bioRxiv and MedRxiv were included to capture emerging evidence in the evolving field of miRNA biomarker research. The searches were conducted from 1 December 2025 to 17 March 2026.

Search strategies were developed independently for each database, accounting for differences in indexing systems and syntax requirements. For PubMed and Embase, MeSH and Emtree vocabulary terms were incorporated alongside keywords to maximise sensitivity. Web of Science and Scopus searches relied on topic-field keyword searching. bioRxiv and medRxiv were searched using basic keywords due to the search bar character limits.

Search strategies were structured around three core concepts: T2D, insulin resistance/HOMA-IR, and circulating miRNA. These were combined using Boolean operators, with additional ethnicity terms for South Asian and European populations included where relevant. Ethnicity-blind versions of the searches were also run to avoid missing relevant studies that did not specify ethnicity in the title/abstract. No date restrictions were applied, and searches were limited to English-language. Full search strategies are provided in Supplementary Text S1.

### 2.4. Selection Process

Database search results were imported into Covidence to remove duplicates and for screening. Screening was conducted in two stages: title and abstract screening, followed by full-text assessment against the pre-specified eligibility criteria. Reasons for full-text exclusion were recorded in Covidence [35], and the study selection process is summarised in the PRISMA 2020 flow diagram.

### 2.5. Data Collection Process

All screening decisions were initially made by the main reviewer (T.K.) and were reviewed by S.S.M. Where data were unclear in the main text, Supplementary Materials were examined, and study authors were contacted for missing information, although no additional data were provided. Screening followed pre-specified criteria entered into Covidence before review to mitigate the risk of inconsistent application of criteria. Both reviewers (T.K. and S.S.M.) reviewed the 14 included studies and confirmed the appropriateness of the final inclusions, data extraction, and quality assessments. The data was extracted using data items relevant to the main aims of the review (Table 2). A blank copy of the form used for data extraction is included in Supplementary Table S1.

### 2.6. Risk of Bias Assessment Methods

The Newcastle–Ottawa Scale was not used in its standard form as it does not adequately capture issues central to this review, particularly miRNA measurement quality, normalisation methods, and ethnicity classification. The adapted domain-based tool was informed by established frameworks: exposure measurement criteria drew on QUADAS-2 principles [36], confounding assessment on ROBINS-E guidance [37], and normalisation quality criteria on published miRNA-specific recommendations [38]. The assessment domains are summarised in Table 3.

Each domain was rated as low, unclear, or high risk of bias, with brief justification recorded in Covidence. Particular emphasis was placed on confounding and HOMA-IR measurement because of their direct relevance to the review question.

### 2.7. Data Synthesis

Findings were synthesised qualitatively, grouped by ethnicity and miRNAs to enable visual comparison across studies in the forest plot. Then, 95% confidence intervals (CIs) were derived for each reported correlation coefficient (Pearson r or Spearman ρ) using Fisher’s z-transformation, calculated as z = ½ ln[(1 + r)/(1 − r)], with SE(z) = 1/√(*n* − 3), back-transformed to the correlation scale [39]. This approximation assumes normality of the Fisher-z statistic for *n* > 10, which all included studies exceeded [40]. Four associations reporting non-correlation metrics (β regression coefficients, tertile comparisons, or percentage change per SD log HOMA-IR) were excluded from the forest plot due to using different scales but were retained in the descriptive information of studies. Significance classification (*p* < 0.05) followed values reported by included studies.

## 3. Results

### 3.1. Study Selection

**Figure 2:**
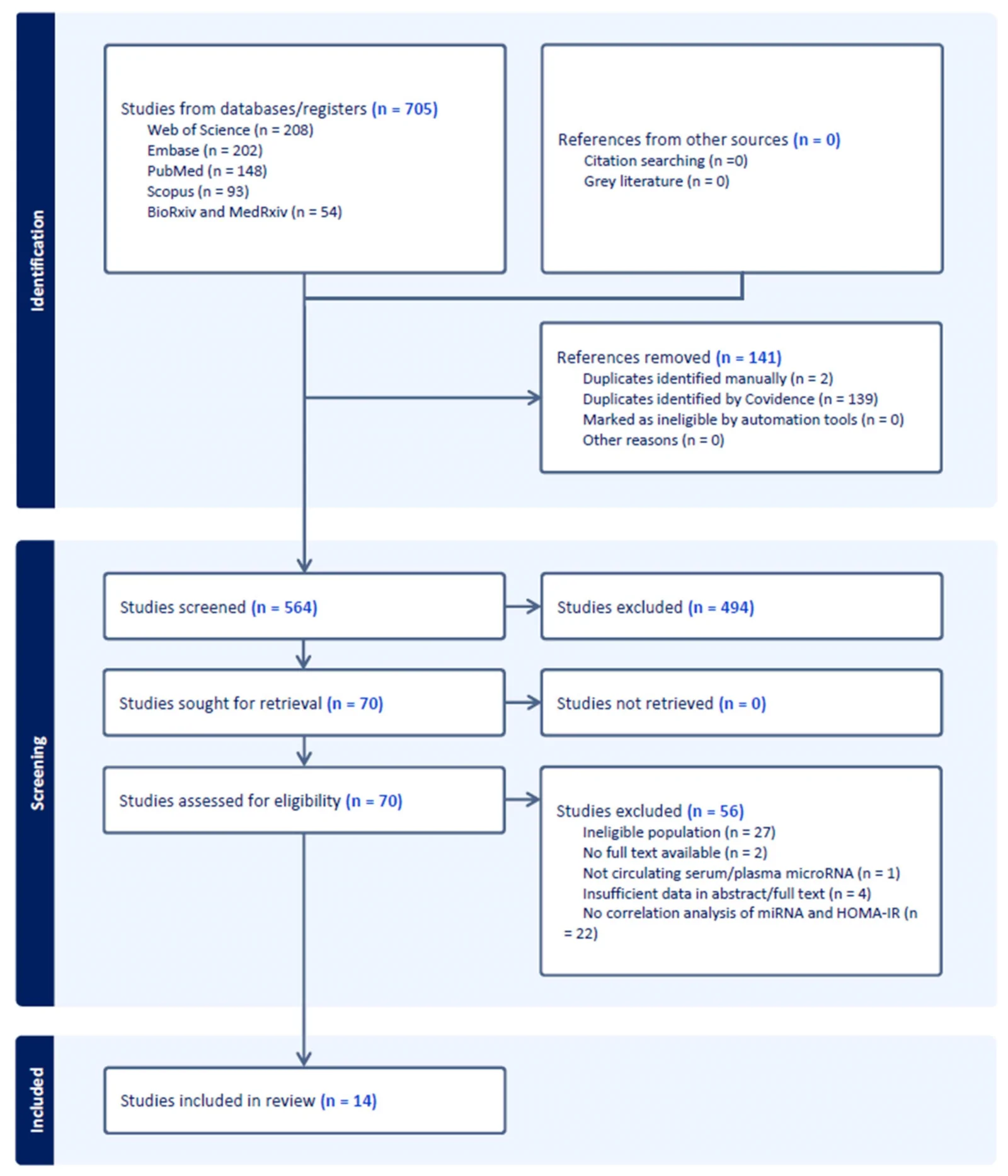
PRISMA 2020 flow diagram of the selection process, created in Covidence.

Database searches identified 705 records (Figure 2). Following the predetermined search criteria and deduplication in Covidence, 564 studies underwent title and abstract screening. Out of these, 494 studies were excluded as clearly ineligible, and 70 were retrieved for full-text review. Following this, 56 studies were excluded, with 14 studies meeting all eligibility criteria for inclusion in the review.

### 3.2. Characteristics of Included Studies

The included studies were published between 2014 and 2024 and are summarised in Table 4. Together, they reported 29 circulating miRNA–HOMA-IR associations across both ethnicities. The predominant study design was cross-sectional case–control (12/14 studies), with one prospective cohort study and one mixed cross-sectional/longitudinal study. Sample sizes varied widely, from 30 to 826 participants, with a median sample size of approximately 111 participants.

Serum and plasma were both used as biological sample types, and all studies measured circulating miRNAs using PCR-based methods. However, normalisation strategies varied substantially, and in some cases, unclear reporting was found. HOMA-IR was used in all studies, but reporting of HOMA version and log-transformation was inconsistent. These differences in normalisation and HOMA-IR handling were important sources of methodological heterogeneity and are considered in the interpretation of findings and associated limitations.

### 3.3. Risk of Bias Assessment

Risk of bias was assessed across seven domains for all fourteen included studies, with results summarised in Table 5. Overall, five studies were judged to be at low risk of bias, five at unclear risk, and four at high risk.

Confounding was the most consistently problematic domain, having scored high risk in 8 out of 14 studies. This was due to failure to adjust for key confounders such as BMI, medication use, and comorbidities like cardiovascular disease. This was particularly important in the Serik et al. studies [45,47,48,49], which recruited participants with coronary artery disease (CAD) alongside T2D, without adjusting for the potential independent effects of CAD on circulating miRNA expression. Additionally, several studies rated high in miRNA Measurement Bias due to the absence of information regarding miRNA isolation procedures, storage conditions, and quality control. Outcome Measurement (HOMA-IR Bias) was rated unclear/high for seven studies, reflecting either failure to report the HOMA model used or information about log-transformation.

Ethnicity classification was another important limitation. Only five studies clearly defined and reported ethnicity, while in the remaining nine studies, ethnicity was inferred from the country of recruitment. This reduced confidence in attributing findings to specific ethnic groups, particularly in cross-ethnic comparisons, as country of residence does not necessarily reflect ethnic background.

### 3.4. Qualitative Synthesis of miRNA–HOMA-IR Associations

**Figure 3:**
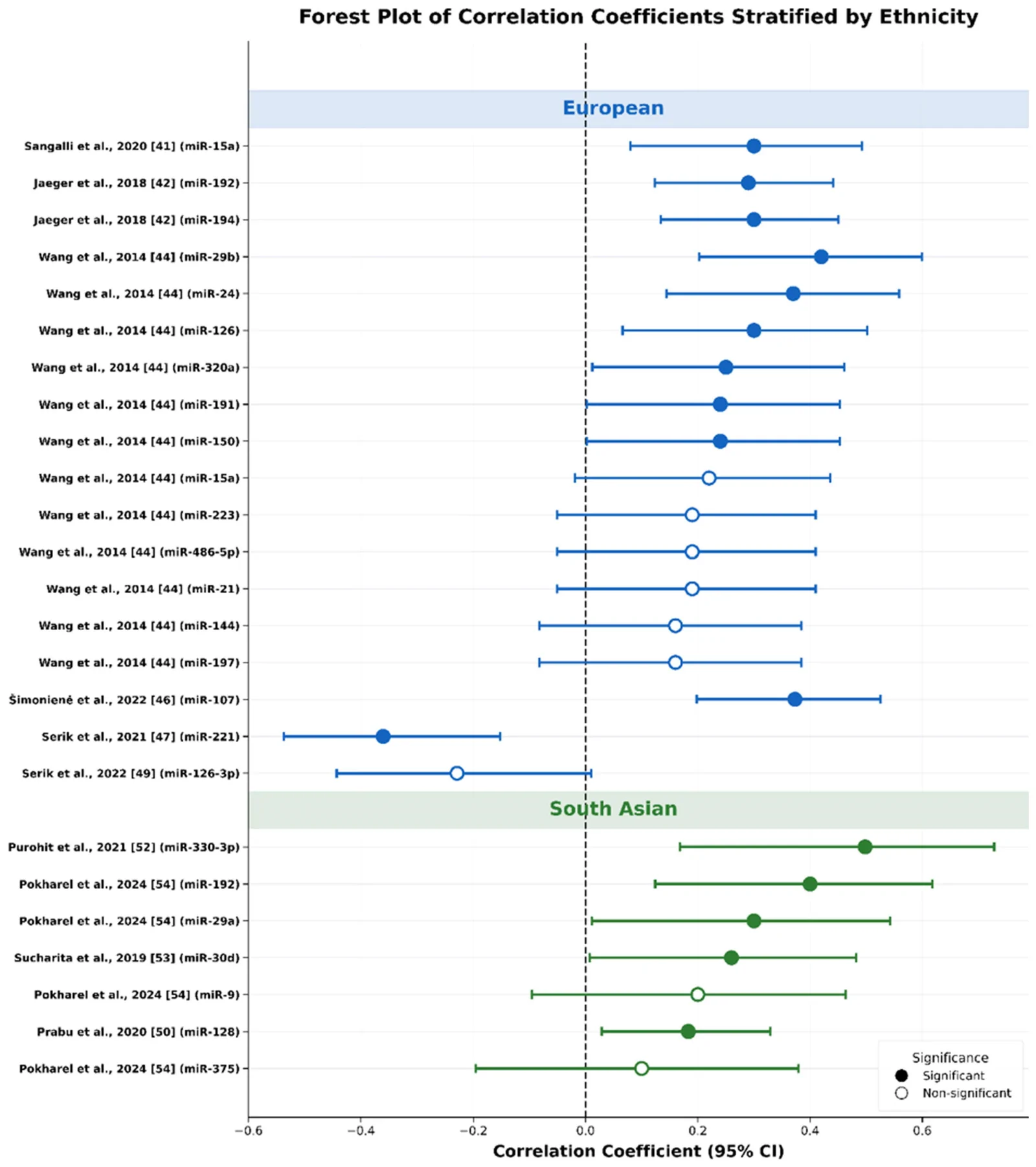
Descriptive forest plot of the 25 associations for which Pearson (r) or Spearman correlation coefficients were reported, allowing visual comparison of effect size and direction across both ethnic groups. Data were from Sangalli et al., 2020 [41], Jaeger et al., 2018 [42], Wang et al., 2014 [44], Šimonienė et al., 2022 [46], Serik et al., 2021 [47], Serik et al., 2022 [49], Prabu et al., 2020 [50], Purohit et al., 2021 [52], Sucharita et al., 2019 [53] and Pokharel et al., 2024 [54].

Across the 14 included studies, 29 circulating miRNA–HOMA-IR associations were reported, involving 21 distinct miRNA species. Most associations were positive, indicating that higher circulating miRNA expression was associated with higher insulin resistance. The main exceptions were miR-126-3p and miR-221, which showed negative associations in the CAD-comorbid studies by Serik and colleagues [45,47,48,49]. A total of 12 of the 29 associations reached statistical significance (*p* < 0.05). In interpreting the findings below, greater weight is given to associations that were statistically significant, derived from methodologically stronger studies, and observed across more than one cohort. Figure 3 presents a descriptive forest plot visualising comparisons of effect sizes and directions across both ethnic groups. The full synthesis of association data is presented in Table 6 and Supplementary Table S2.

### 3.5. miRNAs Reported in European Population

Overall, the European evidence base was mixed in both quality and reliability. The strongest finding was miR-122 in Willeit et al. [43], which stands out because it used a prospective design, included a large sample, log-transformed HOMA-IR, adjusted for covariates, and was the only study in this review to report confidence intervals (CIs). This makes it the most methodologically convincing association identified. The miR-192 and miR-194 findings from Jaeger et al. [42] seem plausible, but they carry less weight because the analyses were unadjusted, and it was unclear how HOMA-IR was handled. Wang et al. [36] reported the highest number of associations from a single European study, although these were unadjusted correlations from a combined cohort, and several did not reach significance. miR-107 in Šimonienė et al. [46] is also worth noting as a positive finding from a low-risk-of-bias study, although the correlation method was not clearly reported. The negative associations for miR-126-3p and miR-221 in the Ukrainian CAD cohorts [45,47,48,49] should be interpreted cautiously, as all were rated high risk of bias, none adjusted for confounding from CAD or related treatment, and two pairs of studies appear to use the same or overlapping cohorts rather than independent samples.

### 3.6. miRNAs Reported in South Asian Populations

Within the South Asian evidence base, Prabu et al. [50] carries the most interpretive weight. It was the only South Asian study rated as low overall risk of bias and reported explicit use of HOMA1, validated spike in normalisation, and a well-characterised population-based cohort. Prabu and colleagues [51] documented the statistically detailed South Asian findings for miR-374a-5p, with similar β coefficients in both unadjusted and sex-adjusted regression models, although confidence was reduced by the high confounding risk rating. The findings for miR-30d by Sucharita et al. [53] and miR-128 by Prabu et al. [50] are supported by reasonably valid methods, but both remain unreplicated. The hsa-miR-330-3p result from Purohit et al.’s study [51] should be treated as provisional because the sample included only 30 participants, methodological reporting was limited, and the study was rated high risk of bias across multiple domains. Pokharel et al.’s [54] study is still useful, especially as the only Nepali study and the only source of cross-ethnic overlap for miR-192 and the miR-29 family, but its findings should also be interpreted cautiously. This is because the correlation coefficients were only available as heatmap approximations, and the sample size of 46 limits confidence in the results.

### 3.7. Cross-Ethnic Comparisons and Patterns

In this review, direct cross-ethnic comparisons of miRNA–HOMA-IR associations were not possible, as no single study reported association data for both South Asian and European participants within the same design. Comparisons can therefore only be drawn at the cross-study level and should be interpreted cautiously.

Despite this, three main descriptive observations are worth documenting. First, miR-192 showed a significant positive association with HOMA-IR in both an independent European study (Jaeger et al. ρ = 0.29, *p* = 0.0007 [42]) and an independent South Asian study (Pokharel et al. ρ ≈ 0.4, *p* < 0.01 [54]). It was the only miRNA found in the literature which is studied in both populations. Second, some members of the miR-29 family also showed positive associations across regions: miR-29b in the European Wang et al. [36] cohort (ρ = 0.42, *p* = 0.0003) and miR-29a in the South Asian Pokharel et al. [54] cohort (ρ ≈ 0.3, *p* < 0.05). Although these are different family members rather than the same miRNA species, the shared positive direction is noteworthy. Third, miR-126-3p showed negative associations in two European studies [45,49] but was not examined in any South Asian studies available.

The majority of miRNAs were investigated in only one ethnic population. For example, miR-122 was reported only in European cohorts, whereas miR-374a-5p was reported only in South Asian cohorts. Therefore, the lack of cross-ethnic overlap may reflect a lack of research studies rather than the absence of evidence.

## 4. Discussion

This systematic review aimed to identify circulating miRNAs associated with insulin resistance, measured by HOMA-IR, in adults with T2D, and to explore whether association patterns are similar or divergent between South Asian and European populations. Across 14 included studies, 29 miRNA–HOMA-IR associations were reported, of which 12 reached statistical significance (*p* < 0.05), and all but four were positively associated.

Overall analysis supported that circulating miRNAs show consistent associations with HOMA-IR in the published literature, with the predominant direction being positive. This is consistent with prior reviews of circulating miRNA dysregulation in T2D more broadly [28], and with mechanistic evidence demonstrating that several of the miRNAs identified here regulate key nodes of the insulin signalling cascade, including components of the PI3K-AKT pathway and IRS proteins [22,26,27].

However, the absence of cross-ethnic data reflects a structural gap in the miRNA biomarker literature. No study directly compared circulating miRNA–HOMA-IR associations between South Asian and European populations, and cross-study comparisons remain limited by differences in study design and methodology. The observed patterns identified here are therefore hypothesis-generating rather than conclusive. The most defensible conclusion is that the literature reveals a substantial gap in cross-ethnic miRNA research, rather than definitive evidence on ethnic differences in circulating miRNA–HOMA-IR associations.

### 4.1. Associations Reported in European Populations: miR-122, miR-192, and miR-194 and “The Hepatic miRNA Cluster”

Together, miR-122, miR-192, and miR-194 may represent a putative hepatic metabolic miRNA signature: three liver-enriched, co-regulated molecules showing positive associations with HOMA-IR across independent European cohorts.

The strongest evidence comes from Willeit et al. [43], who reported a positive association between circulating miR-122 and log HOMA-IR in the Bruneck cohort. Given the study’s low risk of bias and appropriate covariate adjustment, this finding carries substantial weight. miR-122 accounts for approximately 70% of hepatic miRNA content and regulates lipid metabolism, including fatty acid oxidation and cholesterol biosynthesis [55]. Circulating miR-122 increases in hepatocyte stress and NAFLD and correlates with steatosis severity [56], suggesting that its relationship with HOMA-IR likely reflects hepatic lipid overload rather than a direct causal role in insulin resistance.

**Figure 4:**
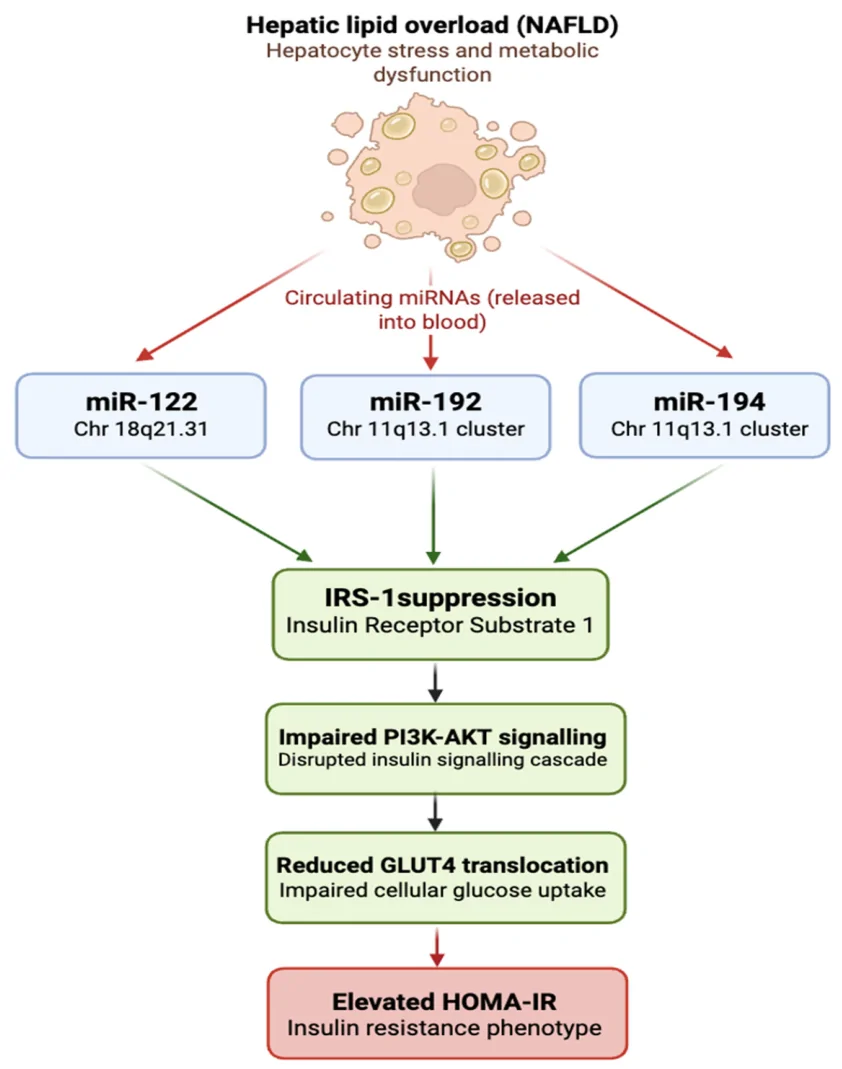
Author-drawn preliminary hypothesis of hepatic metabolic miRNA signature. Hepatic lipid overload associated with NAFLD drives the co-release of three liver-enriched circulating miRNAs: miR-122 and the co-encoded miR-192/miR-194 cluster on chromosome 11q13.1. All three converge on suppression of IRS-1, impairing PI3K-AKT signalling and GLUT4-mediated glucose uptake, providing a mechanistic basis for their co-association with elevated HOMA-IR observed across independent European cohorts in this review.

Jaeger et al. [42] reported significant positive correlations between HOMA-IR and both miR-192 (ρ = 0.29, *p* = 0.0007) and miR-194 (ρ = 0.30, *p* = 0.0005) in an Austrian/UK cohort. These miRNAs are co-encoded at chromosome 11q13.1 and highly expressed in the liver and intestinal epithelium [57], making their concordant upregulation consistent with shared transcriptional control during hepatic metabolic stress. A preliminary hypothesis for consideration is that NAFLD-related hepatocyte dysfunction drives release of miR-122 while activating the miR-192/194 cluster, converging on IRS-1 suppression and impaired PI3K-AKT signalling, thereby increasing HOMA-IR (Figure 4). Although this postulated hepatic signature could provide greater biomarker specificity than individual miRNAs, the findings of Jaeger et al. should be interpreted cautiously because analyses were unadjusted and confidence intervals were not reported. To validate this early hypothesis, studies within larger ethnically defined populations would need to be undertaken.

### 4.2. Associations Reported in South Asian Populations

Prabu et al. [50] reported a significant positive Pearson correlation between circulating miR-128 and HOMA-IR (r = 0.183, *p* = 0.021) in a well-characterised Indian cohort. miR-128 has been reported to target SIRT1, a regulator of insulin sensitivity acting through IRS-1 and FOXO transcription factors [58]. Elevated miR-128 may therefore plausibly contribute to insulin resistance through suppression of SIRT1, although this has not been directly demonstrated in circulating miRNA studies in South Asian populations.

Prabu et al. [51] provided the most statistically informative South Asian result, reporting a positive association between circulating miR-374a-5p and HOMA-IR in both unadjusted (β = 0.109, *p* = 0.013) and sex-adjusted (β = 0.108, *p* = 0.015) models. However, miR-374a-5p remains poorly characterised in insulin resistance currently.

The finding for circulating miR-30d from Sucharita et al. [53] is also relevant to South Asians. miR-30d has been linked to lipid-mediated cell stress and pancreatic β-cell function under lipotoxic conditions [59]. South Asian populations characteristically exhibit greater visceral and ectopic fat deposition even at lower BMI values, contributing to elevated free fatty acid flux and intracellular lipid accumulation in metabolic tissues [11]. This pattern could plausibly influence miR-30d expression and its relationship with insulin resistance, but the evidence remains speculative due to limited sample size and lack of replication. Similarly, the hsa-miR-330-3p association reported by Purohit et al. [52], although statistically significant (ρ = 0.498, *p* = 0.005), should be treated as highly provisional given the very small sample, poor methodological documentation, and assigned high risk of bias.

### 4.3. Cross-Ethnic Signals: The miR-29 Family

The most plausible cross-ethnic signal identified in this review involves the miR-29 family. Wang et al. [44] reported the strongest individual Spearman’s correlation in the European evidence base for miR-29b (ρ = 0.42, *p* = 0.0003) in a Swedish cohort, while Pokharel et al. [54] reported a significant positive association for miR-29a (ρ ≈ 0.3, *p* < 0.05) in a Nepali cohort. miR-29a and miR-29b are members of the miR-29 family, along with miR-29c, encoded from two genomic clusters. All three members share an identical seed sequence (AGCACC), meaning they are predicted to regulate an overlapping set of target genes [60].

Evidence suggests the miR-29 family is mechanistically relevant to insulin resistance. Members of this family have been shown to target IRS-1, a central adaptor protein in the PI3K-AKT insulin signalling pathway, and their overexpression has been associated with impaired insulin-stimulated glucose uptake in adipocytes and attenuation of insulin action in hepatocytes [57,60]. Elevated circulating miR-29 in the context of higher HOMA-IR is therefore consistent with a model in which increased miR-29 expression suppresses IRS-1, disrupts downstream PI3K-AKT signalling, and contributes to reduced cellular insulin sensitivity.

Whether this reflects genuine cross-ethnic consistency or coincidental convergence cannot be resolved from the current data. The two studies differed not only in the miR-29 subgroup examined, but also in methodology, as Pokharel et al. [54] reported coefficients from a heatmap rather than tabulated data without confirmation of exact values. This comparison between miR-29a and miR-29b therefore limits direct cross-ethnic inference, even despite their shared seed sequence. Although the shared seed sequence supports the biological plausibility of similar functional effects, it is not a substitute for direct empirical evidence across ethnic groups. The miR-29 family finding is therefore best characterised as a hypothesis-generating cross-ethnic signal that requires direct investigation in future studies.

### 4.4. Negative Associations: miR-126-3p and miR-221

Serik and colleagues [45,47,48,49] reported negative associations between miR-126-3p and miR-221 and HOMA-IR. All four studied populations had both CAD and T2D. Each was rated as high overall risk of bias; none adjusted for the independent effects of CAD, and two pairs of studies appear to use the same or heavily overlapping cohorts rather than independent samples, so they cannot be treated as independent replications.

The negative directions are not inherently biologically implausible. miR-126-3p is mainly expressed in endothelial cells and is involved in vascular integrity and angiogenic signalling [61]. In advanced cardiovascular disease, circulating miR-126-3p may be reduced because of ongoing endothelial uptake and repair, so lower levels in patients with greater insulin resistance within a CAD cohort may reflect cardiovascular disease burden rather than insulin resistance itself. miR-221 is also involved in vascular smooth muscle cell biology and endothelial function [59,61], and its negative association with HOMA-IR in these cohorts is likewise difficult to separate from CAD severity, medication use, and the bidirectional relationship between CAD and insulin resistance. Without adjustment for these confounders, the associations cannot be attributed specifically to insulin resistance. These findings are therefore treated as exploratory and excluded from the primary synthesis conclusions.

### 4.5. Limitations of This Review and Primary Literature

A limitation of this review is that initial screening was performed by a single reviewer, rather than through independent dual screening. Consequently, the potential for selection bias and inadvertent exclusion or inclusion of studies cannot be entirely excluded, and inter-reviewer agreement could not be formally processed/assessed. However, predefined eligibility criteria within Covidence and prospective registration on OSF were used to standardise decision making and enhance transparency throughout the study selection process.

Study-specific concerns also apply. For Pokharel et al. [54], exact correlation coefficients were unavailable and had to be approximated from a heatmap colour scale, introducing some imprecision. In addition, the four Ukrainian studies [45,47,48,49] appeared to involve substantial cohort overlap, which could not be confirmed from the publications alone and may have led to overrepresentation of CAD-T2D populations in the synthesis.

Several recurring weaknesses in the primary literature also limit confidence in the findings. Most importantly, covariate adjustment was rare: only 3 of the 29 associations reported any adjustments for key factors such as BMI and medication, leaving many reported associations open to confounding.

HOMA-IR reporting was also inconsistent, with only six studies explicitly stating use of HOMA1 and only three confirming log transformation. CIs were reported for only 1 of the 29 associations, limiting direct visual comparison of uncertainty across the evidence base. To partially address this, 95% CIs were derived for plotted associations using Fisher’s z-transformation (Figure 3). This approach was originally derived for Pearson’s r but is conventionally applied to Spearman’s ρ [62]. Small sample sizes in several South Asian studies (*n* = 30–46) produced wide intervals that should be interpreted with caution. The derived CIs therefore provide an indicative uncertainty range rather than a definitive statistical inference.

Variation in miRNA laboratory measurements and normalisation methods introduce further technical heterogeneity and limitations which should be considered carefully in interpretation of results [38].

Ethnicity was not explicitly reported in nine studies and was therefore inferred from the country of recruitment. This introduces the potential for population misclassification, as country of residence does not necessarily reflect ethnic background. Consequently, the validity of cross-ethnic comparisons should be interpreted with caution, as some observed differences may reflect inaccuracies in population assignment rather than true ethnic variation.

The cross-ethnic findings should also be interpreted cautiously. Although several miRNAs appeared to show stronger associations in South Asian populations, many of these observations were derived from a small number of studies with relatively modest sample sizes. Furthermore, ethnicity was not consistently reported across the evidence base and, in some cases, had to be inferred from the country of recruitment. Given the ethnic heterogeneity present within many countries, this may have resulted in some misclassification of study populations. Consequently, apparent differences between South Asian and European cohorts may partly reflect limitations in study reporting and population assignment rather than true biological variation. The observed ethnic patterns are therefore best regarded as hypothesis-generating and warrant confirmation in larger, well-characterised cohorts with consistently reported ethnicity and standardised methodological approaches (refer to Future Research Directions and Wider Implications).

Additionally, the median sample size was approximately 111, below what is often considered desirable for biomarker correlation studies, increasing the risk of unstable estimates and chance findings.

### 4.6. Clinical Implications

Despite the limitations outlined above, the predominance of positive circulating miRNA–HOMA-IR associations provides preliminary support for circulating miRNAs as minimally invasive biomarkers of insulin resistance. Their stability in biological fluids, accessibility through routine blood sampling, and compatibility with RT-qPCR platforms support their potential feasibility for metabolic risk assessment [25,28], but caution is warranted here as the abundance of miRNA in circulation does not necessarily mean that its expression is also increased in specific cells or tissues or disease state.

The potential for ethnically specific miRNA biomarker profiles is particularly relevant to South Asian populations. Conventional metabolic risk algorithms, including those based on BMI thresholds and standard glycaemic parameters, are less sensitive for identifying T2D risk in South Asians. Ethnically calibrated circulating miRNA signatures could potentially complement existing risk stratification tools by capturing molecular features of metabolic risk that are not reflected in standard anthropometric or glycaemic measures. The associations of miR-374a-5p and miR-128 with HOMA-IR in South Asian cohorts provide a preliminary indication that such ethnically distinct molecular profiles may exist, though the evidence is currently insufficient to support clinical translation. Validation in large, ethnically stratified cohorts is essential before clinical application could be considered.

### 4.7. Future Research Directions and Wider Implications

The main research priority arising from this review is the design of adequately powered, prospective, multi-ethnic studies that include both South Asian and European participants within the same design, allowing direct comparison of circulating miRNA–HOMA-IR associations by ethnicity. These studies should pre-specify candidate miRNAs based on the current evidence, including miR-122, miR-192, the miR-29 family, miR-374a-5p, and miR-128, and should be powered to detect ethnicity-by-miRNA interaction effects rather than main effects alone.

Methodological standardisation is equally important. More consistent HOMA-IR reporting, including explicit statement of model version (HOMA1 or HOMA2), log transformation, and covariate adjustment for at least age, sex, BMI, and medication use, would reduce the heterogeneity that currently limits comparison across studies. Standardised normalisation protocols, for example, spike-in controls combined with validated endogenous reference miRNAs selected using GeNorm or NormFinder algorithms, would also improve technical reproducibility and inter-laboratory comparability [38].

Three replication priorities emerge. First, the positive miR-192 association with HOMA-IR, reported in independent South Asian and European studies, should be tested in a directly comparative multi-ethnic design. Second, the miR-29 family should be examined across both ethnic groups to establish whether the same species shows consistent positive associations and whether these reflect shared or distinct pathways. Third, the hepatic metabolic miRNA signature (miR-122, miR-192, miR-194) should be validated as a coordinated panel in a prospective multi-ethnic study. An ideal design would recruit matched South Asian and European participants from an established cohort such as UK Biobank, measuring all three miRNAs alongside MRI-derived proton density fat fraction (MRI-PDFF) as a gold-standard measure of hepatic steatosis. This would directly test whether the three miRNAs co-vary with liver fat across ethnic groups and whether the signature is associated within European populations or generalises to South Asians, where NAFLD prevalence and hepatic fat distribution differ phenotypically. To finalise these judgements and justify the prioritisation of miRNA candidates for future research, Table 7 presents a GRADE-adapted certainty of evidence assessment across the five priority targets identified in this review [63].

The fragmented literature identified in this review reflects not only scientific uncertainty but also the structural underrepresentation of South Asian populations in biomedical research. South Asians make up around 25% of the global population but fewer than 2% of participants in genetic and genomic studies [29]. A similar imbalance appears in the miRNA biomarker literature, with five South Asian studies identified compared with nine European studies, despite the greater T2D burden in South Asians. If circulating miRNA biomarkers are developed and validated mainly in European cohorts, they may perform poorly or inequitably when applied to South Asian patients, where earlier and more sensitive risk detection is especially needed. This translational risk should be addressed explicitly in future biomarker development programmes.

A related concern is that normalisation strategies, reference gene panels, and population-level miRNA expression data have largely been established in European-ancestry studies, raising doubts about their suitability for South Asian samples. Because population-specific genetic variation in miRNA gene sequences and regulatory regions can influence miRNA expression [30], future studies should validate normalisation approaches within each ethnic cohort rather than assume cross-ethnic transferability.

Finally, future research should move beyond observational biomarker studies towards mechanistic work explaining why miRNA-insulin resistance associations may differ across ethnic groups. Integrating circulating miRNA profiling with measures of visceral adiposity, ectopic fat deposition, and genetic variation in miRNA regulatory loci within multi-ethnic cohorts would help disentangle the contributions of biology, environment, and measurement. That would move the field beyond merely describing ethnic differences towards explaining them, which is the more important scientific and clinical goal.

## 5. Conclusions

This is the first systematic review to specifically examine and compare circulating miRNA signatures associated with insulin resistance, measured by HOMA-IR, across South Asian and European study populations. Across 14 included studies and 29 reported associations, circulating miRNAs showed predominantly positive associations with HOMA-IR. The most robust individual finding was the positive association between circulating miR-122 and log HOMA-IR in the Bruneck prospective cohort, in line with the recognised role of miR-122 as a hepatic metabolic biomarker. Co-directional associations for miR-192 and miR-194 in an independent European cohort further suggest a putative hepatic miRNA cluster linked to insulin resistance in European populations, although this remains to be replicated with appropriately adjusted analyses.

miR-192 and the miR-29 family were the most plausible cross-ethnic signals identified, but direct within-study ethnic comparison was not possible, and most miRNAs were investigated in only one ethnic population. This reflects a substantial gap in the literature rather than evidence of true ethnic differences in miRNA–HOMA-IR associations. The clinical and scientific implications of the findings underline the need for multi-ethnic, adequately powered, and methodologically standardised studies in this field. This review provides a mapped evidence base and highlights priority targets for future cross-ethnic research, particularly miR-122, miR-192, the miR-29 family, miR-128, and miR-374a-5p.

## Figures and Tables

**Table 1:** PICOS eligibility criteria for study inclusion and exclusion.

Domain	Inclusion Criteria	Exclusion Criteria
**Population**	Adults ≥ 18 years; diagnosed with T2DM, pre-diabetes, or insulin resistance; South Asian ethnicity and/or European ethnicity; ethnicity clearly stated or inferable from study description.	Paediatric populations (<18 years); type 1 diabetes mellitus; gestational diabetes; animal or in vitro studies.
**Intervention/Exposure**	Circulating cell-free miRNA measured in human serum or plasma; quantified using molecular techniques (RT-qPCR, TaqMan, SYBR Green, or NanoString).	PBMC-derived or intracellular miRNA; miRNA from tissue biopsies; miRNA gene polymorphism or SNP studies; in silico or bioinformatic-only analyses.
**Comparator/Context**	Statistical framework permitting assessment of miRNA-insulin resistance association: correlation analysis (Pearson, Spearman, regression) or group comparison by insulin resistance status (e.g., high vs. low HOMA-IR).	Studies reporting only T2DM vs. control differential expression without insulin resistance analysis; studies assessing only glycaemic measures (HbA1c, fasting glucose) without insulin resistance outcome; no direct statistical association between miRNA and insulin resistance.
**Outcome**	Primary outcome: insulin resistance quantified by HOMA-IR. Direct statistical association between circulating miRNA expression and HOMA-IR (e.g., Pearson/Spearman correlation, regression analysis, or HOMA-IR-stratified group comparison).	Studies reporting only differential miRNA expression between T2DM and controls without insulin resistance analysis; studies using glycaemic outcomes only (HbA1c, fasting glucose) without HOMA-IR or fasting insulin.
**Study design**	Observational designs: cross-sectional, case–control, cohort, prospective cohort. RCTs if baseline miRNA–insulin resistance association is reported. English-language publications. Original human data.	RCTs where no baseline miRNA–insulin resistance association is reported; mechanistic adipose tissue studies without circulating miRNA data; review articles; case reports; conference abstracts without extractable data.

**Table 2:** Data items extracted from each included study.

Domain	Data Item	Format/Detail Recorded
**Study & participant characteristics**	First author; publication year; country	Text
	Study design	Cross-sectional/case–control/cohort/prospective cohort
	Sample size	*n* (total and per group)
	Ethnic group and ethnicity classification method	Self-reported/registry/unclear
	T2D diagnosis criteria; recruitment source	Hospital clinic/community/population registry
	Age; sex distribution; BMI; T2DM duration	Mean ± SD; % male/female; kg/m^2^; years
	Medication use	Metformin/insulin/other/not reported
	Funding source; declared conflicts of interest	Text
**Biological & methodological variables**	Biological sample type	Serum/plasma
	Sample collection and storage protocol	Described/not described
	miRNA quantification platform	RT-qPCR/TaqMan/SYBR Green/microarray/sequencing
	Normalisation method	Reference gene (e.g., U6, miR-16)/spike-in (Cel-miR-39)/global mean/not reported
	miRNA(s) investigated; direction of expression change	miRNA name; positive/negative/null
**Outcome & statistical data**	HOMA-IR values reported	Mean/median/group-stratified; HOMA1/HOMA2/unclear; log-transformed (yes/no)
	Validation cohort; RNA extraction method	Yes/No
	Statistical method for miRNA–HOMA-IR association	Pearson/Spearman correlation/linear regression/group comparison
	Effect estimate and precision	*r* or ρ; β coefficient; OR/HR; group comparison *p*-value
	95% confidence intervals	Reported/not reported
	Covariates adjusted for	Age/sex/BMI/medication use/other

**Table 3:** Domains included in the adapted risk of bias assessment tool.

Domain	What Was Assessed
**Selection bias**	Representativeness of the study population and recruitment method
**Exposure measurement (miRNA measurement bias)**	Adequacy of miRNA isolation, quantification platform, and normalisation protocol
**Outcome measurement (HOMA-IR bias)**	Validity and completeness of HOMA-IR reporting, including log-transformation and HOMA version used (HOMA1, HOMA2, or unclear)
**Confounding**	Whether key confounders, including age, sex, BMI, and medication use, were identified and adjusted for
**Statistical reporting**	Completeness of statistical reporting, including effect estimates, confidence intervals, *p*-values, and multiple testing correction
**Selective reporting**	Risk of selective reporting of miRNA findings
**Ethnicity classification bias**	Clarity and rigour of ethnic group definition and method of ethnicity ascertainment, such as self-report, registry-based classification, or unclear methods

**Table 4:** Summary of included studies’ characteristics (*n* = 14).

Study (Year)	Country	Study Design	*n*	Sample Type	miRNA Platform	Normalisation Method	HOMA-IR Reporting
Sangalli et al., 2020 [41]	Italy	Cross-sectional	76	Plasma	TaqMan RT-qPCR (QuantStudio 6)	Exogenous spike-in (Cel-miR-39)	Yes; version unclear; not log-transformed
Jaeger et al., 2018 [42]	Austria; UK	Cross-sectional & longitudinal	129 (cross-sectional); 213 (longitudinal)	Serum	Exiqon qRT-PCR arrays + individual qRT-PCR	Global mean (discovery); GeNorm/NormFinder (validation: miR-103, miR-106a, miR-425)	Yes; version unclear; log status unclear
Willeit et al., 2017 [43]	Italy; UK; Ireland; Nordic countries	Prospective cohort	826 (810 with miRNA data)	Serum & plasma	TaqMan qRT-PCR	Unclear for biofluids (U6 + Cel-miR-39 stated for cell/tissue; not confirmed for serum)	Yes; HOMA1; log-transformed (Log HOMA-IR)
Wang et al., 2014 [44]	Sweden	Cross-sectional	68	Plasma	qRT-PCR (miRNeasy/QIAzol)	miR-425 (single reference gene; selected by GeNorm/NormFinder)	Yes; version unclear; log-transformed
Serik et al., 2023 [45]	Ukraine	Cross-sectional	111 (68 CAD+T2DM; 25 CAD; 18 controls)	Plasma	Real-time PCR	Not reported	Yes; version/log status not reported
Šimonienė et al., 2022 [46]	Lithuania	Cross-sectional	108 (53 T2DM; 54 controls)	Serum	TaqMan qRT-PCR	Not reported explicitly	Yes; HOMA1-style; not log-transformed
Serik et al., 2021 [47]	Ukraine	Cross-sectional	80 (58 CAD+T2DM; 22 CAD)	Plasma	Real-time PCR	Not reported explicitly	Yes; version/log status not reported
Serik et al., 2020 [48]	Ukraine	Cross-sectional	80 (58 CAD+T2DM; 22 CAD) + 20 healthy	Unclear (circulating)	Real-time PCR	U6 small nuclear RNA	Yes; version/log status not reported
Serik et al., 2022 [49]	Ukraine	Cross-sectional	111 (68 CAD+T2DM; 25 CAD; 18 controls)	Plasma	Real-time PCR	U6 small nuclear RNA	Yes; version/log status not reported
Prabu et al., 2020 [50]	India	Cross-sectional	160 (40 per group)	Serum	SYBR Green qRT-PCR (miRCURY LNA)	Spike-in Cel-miR-39-3p	Yes; HOMA1; not log-transformed
Prabu et al., 2015 [51]	India	Cross-sectional	145 (NGT 49; IGT 47; T2DM 49)	Serum	miRCURY LNA qRT-PCR (Exiqon, LightCycler 480)	Global mean normalisation (discovery); cel-miR-39 spike-in (validation)	Yes; HOMA1; not log-transformed
Purohit et al., 2021 [52]	India	Cross-sectional	30 (15 T2DM; 15 controls)	Serum	Real-time PCR	RNU6 housekeeping gene	Yes; version/log status not reported
Sucharita et al., 2019 [53]	India	Cross-sectional	60 (T2DM 30; control 30)	Plasma	TaqMan qRT-PCR (Bio-Rad CFX, run in duplicate)	miR-16 endogenous reference gene	Yes; HOMA1-style; not log-transformed
Pokharel et al., 2024 [54]	Nepal	Cross-sectional	46 (25 controls; 9 prediabetes; 12 T2DM)	Plasma	RT-qPCR (Azure Cielo DX system)	RNU6B reference gene	Yes; HOMA1; not log-transformed

Legend: CAD = coronary artery disease; NGT = normal glucose tolerance; IGT = impaired glucose tolerance; T2DM = type 2 diabetes mellitus; qRT-PCR = quantitative reverse transcription PCR; HOMA-IR = Homeostatic Model Assessment of Insulin Resistance. Wang 2014 [44] recruited both Swedish-born and Iraqi-born participants; only Swedish-born data were extracted as Iraq is a Middle Eastern country.

**Table 5:** Risk of bias summary for included studies (*n* = 14).

Study (Year)	Selection Bias	Exposure Measurement (miRNA)	Outcome Measurement (HOMA-IR)	Confounding	Statistical Reporting	Selective Reporting	Ethnicity Classification	Overall
Sangalli et al., 2020 [41]	Low	Low	Unclear	Unclear	Low	Unclear	Unclear	Unclear
Jaeger et al., 2018 [42]	Unclear	Low	Unclear	High	Unclear	Low	Unclear	Unclear
Willeit et al., 2017 [43]	Low	Low	Low	Low	Low	Low	Unclear	Low
Wang et al., 2014 [44]	Low	Low	Unclear	Unclear	Unclear	Low	Low	Low
Serik et al., 2023 [45]	Unclear	Unclear	High	High	Unclear	Unclear	Unclear	High
Šimonienė et al., 2022 [46]	Low	Low	Low	Low	Unclear	Low	Unclear	Low
Serik et al., 2021 [47]	Unclear	High	Low	High	Unclear	High	Unclear	High
Serik et al., 2020 [48]	Unclear	High	High	High	Unclear	Unclear	Unclear	High
Serik et al., 2022 [49]	Unclear	Unclear	Unclear	High	Low	Unclear	Unclear	Unclear
Prabu et al., 2020 [50]	Low	Low	Low	Low	Low	Low	Low	Low
Prabu et al., 2015 [51]	Low	Low	Low	High	Unclear	Unclear	Low	Unclear
Purohit et al., 2021 [52]	High	Unclear	Unclear	High	Unclear	Unclear	Unclear	High
Sucharita et al., 2019 [53]	Unclear	Low	Low	Unclear	Unclear	Low	Low	Low
Pokharel et al., 2024 [54]	Low	Low	Low	High	High	Low	Low	Unclear

Domains: Selection Bias; Exposure Measurement (miRNA Measurement Bias); Outcome Measurement (HOMA-IR Bias); Confounding; Statistical Reporting; Selective Reporting; Ethnicity Classification Bias. Overall rating reflects pattern of domain ratings with greatest weight given to Confounding and Outcome Measurement. These directly affect the validity of the miRNA–HOMA-IR association due to inadequate adjustment for factors, which could distort the observed relationship, and unclear HOMA-IR calculation, model choice or transformation, which directly affects the accuracy and comparability of the insulin-resistance outcome.

**Table 6:** Summary of circulating miRNA–HOMA-IR associations across included studies (*n* = 29 associations from 14 studies).

Study (Year)	miRNA	Direction	Effect Size	*p*-Value
European Studies				
Sangalli et al., 2020 [41]	miR-15a	Positive	ρ = 0.30	0.003
Jaeger et al., 2018 [42]	miR-194	Positive	ρ = 0.30	0.0005
Jaeger et al., 2018 [42]	miR-192	Positive	ρ = 0.29	0.0007
Willeit et al., 2017 [43]	miR-122	Positive	+67% per 1 SD log HOMA-IR (95% CI: 39–101%)	<0.001
Wang et al., 2014 [44]	miR-29b	Positive	ρ = 0.42	0.0003
Wang et al., 2014 [44]	miR-24	Positive	ρ = 0.37	0.002
Wang et al., 2014 [44]	miR-126	Positive	ρ = 0.30	0.01
Wang et al., 2014 [44]	miR-191	Positive	ρ = 0.24	0.04
Wang et al., 2014 [44]	miR-320a	Positive	ρ = 0.25	0.04
Wang et al., 2014 [44]	miR-150	Positive	ρ = 0.24	0.05
Wang et al., 2014 [44]	miR-21	Positive	ρ = 0.19	0.12
Wang et al., 2014 [44]	miR-144	Positive	ρ = 0.16	0.19
Wang et al., 2014 [44]	miR-197	Positive	ρ = 0.16	0.19
Wang et al., 2014 [44]	miR-223	Positive	ρ = 0.19	0.11
Wang et al., 2014 [44]	miR-486-5p	Positive	ρ = 0.19	0.13
Wang et al., 2014 [44]	miR-15a	Positive	ρ = 0.22	0.07
Serik et al., 2023 [45]	miR-126-3p	Negative	Not reported (tertile comparison)	0.041
Šimonienė et al., 2022 [46]	miR-107	Positive	r = 0.373	0.035
Serik et al., 2021 [47]	miR-221	Negative	r = −0.36	0.006
Serik et al., 2020 [48]	miR-221	Negative	3rd tertile: 0.41 vs. 1st: 0.70	0.028
Serik et al., 2022 [49]	miR-126-3p	Negative	r = −0.229	0.041
South Asian Studies				
Prabu et al., 2020 [50]	miR-128	Positive	r = 0.183	0.021
Prabu et al., 2015 [51]	miR-374a-5p	Positive	β = 0.108	0.015
Purohit et al., 2021 [52]	hsa-miR-330-3p	Positive	ρ = 0.498	0.005
Sucharita et al., 2019 [53]	miR-30d	Positive	r = 0.26	0.04
Pokharel et al., 2024 [54]	miR-192	Positive	ρ ≈ 0.4 (heatmap)	<0.01
Pokharel et al., 2024 [54]	miR-29a	Positive	ρ ≈ 0.3 (heatmap)	<0.05
Pokharel et al., 2024 [54]	miR-9	Positive	ρ ≈ 0.2 (heatmap)	>0.05
Pokharel et al., 2024 [54]	miR-375	Positive	ρ ≈ 0.1 (heatmap)	>0.05

Key: *p*-values colour coded for visual ease: red = non-significant (*p* > 0.05); light green = significant (*p* < 0.05); dark green = highly significant (*p* < 0.01). Full details are presented in Supplementary Table S3.

**Table 7:** GRADE-adapted certainty of evidence for priority miRNA–HOMA-IR candidates for future research.

miRNA	Studies (*n*)	Risk of Bias	Consistency	Precision	Ethnic Coverage	Certainty
miR-122	1 (*n* = 826)	Low	n/a	Moderate	European only	Moderate
miR-192	2 (*n* = 129; *n* = 46)	Unclear	Consistently positive	Low	European + South Asian	Low
miR-29 family	2 (*n* = 68; *n* = 46)	Low/Unclear	Consistently positive (different members of family)	Moderate	European + South Asian (indirect)	Low
miR-128	1 (*n* = 160)	Low	n/a	Low	South Asian only	Very low
miR-374a-5p	1 (*n* = 146)	High	n/a	n/a	South Asian only	Very low

Table 4 shows certainty ratings: Moderate = moderately confident effect is real but needs further research; Low = further research likely to change confidence in estimate; Very low = true effect is uncertain (evidence base shows promise but is too weak currently). Precision domain was determined by how wide the CI is relative to the reported effect: High = roughly ±25% of the estimate; Moderate: roughly ±25–50% of the estimate; Low = wider than ±50% of the estimate. miR-374a-5p used a β regression coefficient, therefore, units were not standardised for Fisher’s z-transformation for CIs, so precision verdict was not possible.

## Data Availability

No new data were created or analyzed in this study. Data sharing is not applicable to this article.

## Supplementary Materials

The following supporting information can be downloaded at: https://www.mdpi.com/article/10.3390/ijms27219827/s1. References [41,42,43,44,45,46,47,48,49,50,51,52,53,54] are cited in the Supplementary Materials.

## Abbreviations

The following abbreviations are used in this manuscript:

T2D	Type 2 diabetes mellitus
miRNA	MicroRNAs
IRS	Insulin Resistance
IGT	Impaired glucose tolerance
eQTLs	Expression quantitative trait locus
