# Supplementary material for: Circulating microRNAs Associated with Insulin Resistance in South Asian and European Adults with Type 2 Diabetes (T2D): A Systematic Review and Mapping of Ethnic Patterns
Source: Int J Mol Sci. 2026 Nov 2;27(21):9827. doi: 10.3390/ijms27219827 (PMC13763550; doi:10.3390/ijms27219827)
Supplement: Supplementary file 1 [file ijms-27-09827-s001.zip › ijms-4531698-supplementary.pdf]

Supplementary Tables: IJMS paper

Circulating microRNAs associated with insulin resistance in South Asian and European adults with type 2 diabetes: A systematic review and mapping of ethnic patterns.

Supplementary Text S1: Full Database Search Strategies

The following search strategies were used to identify eligible studies across six electronic databases. All searches were conducted by the 17.03.2026. For PubMed, Embase and Web of Science, both a combined ethnicity search and an ethnicity-blind parallel search were conducted. No date limits were applied and language was restricted to English where the database interface allowed this filter.

A.1 PubMed

Combined ethnicity search

(("Type 2 diabetes" OR "Type II diabetes" OR T2D OR "non-insulin dependent diabetes" OR prediabetes OR "impaired glucose tolerance" OR "impaired fasting glucose" OR "diabetes mellitus, type 2"[MeSH])) AND ("insulin resistance" OR "insulin sensitivity" OR HOMA-IR OR "homeostatic model assessment" OR "hyperinsulinemic clamp" OR "euglycemic clamp" OR "fasting insulin" OR OGTT OR "oral glucose tolerance test" OR "insulin signalling" OR "insulin resistance"[MeSH]) AND (microRNA* OR miRNA* OR "miR-" OR "circulating microRNA" OR "serum microRNA" OR "plasma microRNA" OR "non-coding RNA" OR "MicroRNAs"[MeSH]) AND ("South Asian" OR India* OR Indian* OR Pakistan* OR Banglades* OR "Sri Lanka*" OR "Asian continental ancestry group"[MeSH] OR "European continental ancestry group"[MeSH] OR Europe* OR "White" OR Caucasian*)

Ethnicity-blind parallel search

(("Type 2 diabetes" OR "Type II diabetes" OR T2D OR "non-insulin dependent diabetes" OR prediabetes OR "impaired glucose tolerance" OR "impaired fasting glucose" OR "diabetes mellitus, type 2"[MeSH])) AND ("insulin resistance" OR "insulin sensitivity" OR HOMA-IR OR "homeostatic model assessment" OR "hyperinsulinemic clamp" OR "euglycemic clamp" OR "fasting insulin" OR OGTT OR "oral glucose tolerance test" OR "insulin signalling" OR "insulin resistance"[MeSH]) AND (microRNA* OR miRNA* OR "miR-" OR "circulating microRNA" OR "serum microRNA" OR "plasma microRNA" OR "non-coding RNA" OR "MicroRNAs"[MeSH])

A.2 Embase

Combined ethnicity search

('type 2 diabetes mellitus'/exp OR 'type 2 diabetes' OR 'type II diabetes' OR T2D OR prediabetes OR 'impaired glucose tolerance' OR 'impaired fasting glucose') AND ('insulin resistance'/exp OR 'insulin resistance' OR 'insulin sensitivity' OR 'homeostasis model assessment' OR HOMA-IR OR 'fasting insulin' OR 'oral glucose tolerance test' OR OGTT OR 'hyperinsulinemic clamp') AND ('microrna'/exp OR microrna* OR mirna* OR 'mir-' OR 'circulating microrna' OR 'serum microrna' OR

'plasma microrna' OR 'non coding rna') AND ('south asian'/exp OR 'asian continental ancestry group' OR india* OR indian* OR pakistan* OR bangladesh* OR 'sri lanka*' OR 'european continental ancestry group' OR europe* OR caucasian* OR 'white population')

Ethnicity-blind parallel search

('type 2 diabetes mellitus'/exp OR 'type 2 diabetes' OR 'type ii diabetes' OR t2d OR prediabetes OR 'impaired glucose tolerance' OR 'impaired fasting glucose') AND ('insulin resistance'/exp OR 'insulin resistance' OR 'insulin sensitivity' OR 'homeostasis model assessment' OR 'homa ir' OR 'fasting insulin' OR 'oral glucose tolerance test' OR ogtt OR 'hyperinsulinemic clamp') AND ('microrna'/exp OR microrna* OR mirna* OR 'mir-' OR 'circulating microrna' OR 'serum microrna' OR 'plasma microrna' OR 'non coding rna') AND ([adult]/lim OR [young adult]/lim OR [middle aged]/lim OR [aged]/lim) AND [humans]/lim AND [english]/lim AND 'homa ir'

A.3 Web of Science

Combined ethnicity search

TS=("type 2 diabetes" OR T2D OR prediabet*) AND TS=("insulin resistance" OR "fasting insulin" OR "homeostasis model assessment") AND TS=(microRNA* OR miRNA* OR miR*) AND TS=("South Asian*" OR Indian* OR Pakistani* OR Bangladeshi* OR "Sri Lankan*" OR European* OR Caucasian* OR White*)

Irrelevant countries excluded parallel search

(microRNA* OR miRNA* OR "micro RNA*" OR "miR-*") AND ("insulin resistance" OR HOMA-IR OR "homeostasis model assessment") AND ("type 2 diabetes" OR T2DM OR "diabetes mellitus type 2") and Article (Document Types) and English (Languages) and PEOPLES R CHINA or USA or IRAN or EGYPT or SOUTH KOREA or BRAZIL or AUSTRALIA or JAPAN or SAUDI ARABIA or CANADA or MEXICO or IRAQ or RUSSIA or NEW ZEALAND or SOUTH AFRICA or ECUADOR or THAILAND or QATAR or ARGENTINA or CAMEROON or ISRAEL or MOROCCO or NEPAL or PHILIPPINES or U ARAB EMIRATES (Exclude – Countries/Regions)

Note: The Web of Science ethnicity-blind search applied country/region exclusion filters to focus on regions with South Asian and European populations, as the full ethnicity-blind search gave 914 results.

A.4 Scopus

Combined ethnicity search

("type 2 diabetes" OR "type II diabetes" OR T2D OR "non-insulin dependent diabetes" OR prediabet* OR "impaired glucose tolerance" OR "impaired fasting glucose") AND ("insulin resistance" OR "insulin sensitivity" OR HOMA-IR OR "homeostasis model assessment" OR "homeostatic model assessment" OR "hyperinsulinemic clamp" OR "euglycemic clamp" OR "fasting insulin" OR "oral glucose tolerance test" OR OGTT OR "insulin signalling" OR "insulin signaling" OR "IRS-1" OR "PI3K-AKT pathway") AND (microRNA* OR miRNA* OR miR* OR "circulating microRNA" OR "serum microRNA" OR "plasma microRNA" OR "cell-free microRNA" OR "non-coding RNA" OR ncRNA*) AND ("South Asian" OR "South Asians" OR India* OR Indian* OR Pakistan* OR Banglades* OR "Sri Lanka" OR "Sri Lankan" OR Nepal* OR Europe* OR Caucasian* OR "White population" OR "European ancestry" OR "United Kingdom" OR UK OR Britain OR British OR Ireland OR Irish OR France OR French OR Germany OR German OR Netherlands OR Dutch OR Belgium OR Belgian OR Switzerland OR Swiss OR Austria OR Austrian OR Sweden OR Swedish OR Norway OR Norwegian OR Denmark OR Danish OR Finland OR Finnish OR Italy OR Italian OR Spain OR Spanish OR Portugal OR Portuguese OR Greece OR Greek)

Note: Scopus was capable of including all relevant countries in the main combined search term, so all irrelevant countries were screened out.

A.5 bioRxiv and medRxiv

type 2 diabetes OR T2D OR type II diabetes AND insulin resistance OR HOMA-IR AND microRNA* OR miR*

Note: bioRxiv and medRxiv do not support MeSH terms, controlled vocabulary, or advanced Boolean filtering. A simplified keyword search was used. Ethnicity terms were not applied given the limited search functionality of these platforms. Results were screened against full eligibility criteria.

Supplementary Table S1: Blank Data Extraction Template

The following template was developed prior to data extraction and entered into Covidence. It was applied to all 14 included studies.

**Identification**	
**Field**	**Extracted data**
Country	
Setting (hospital clinic / community / registry)	
Author name(s)	
Conflict of interest reported? (Yes/No)	
Ethnicity definition method (self-reported / registry / unclear)	
Funding source	
Recruitment source	
World region (Europe / South Asia)	
Year of publication	
Notes	
**Methods**	
**Field**	**Extracted data**
Study design	
Adjusted analyses performed? (Yes/No)	
Biological sample type (serum / plasma)	
Collection protocol described? (Yes/No)	
Covariates adjusted for	
miRNA quantification platform (qRT-PCR / microarray / sequencing)	
Normalisation method (U6 / spike-in / global mean / unclear)	

Observational type (cross-sectional / longitudinal / case-control)	
RNA extraction method	
Validation cohort used? (Yes/No)	
Notes	
**Population**	
**Field**	**Extracted data**
Inclusion criteria	
Exclusion criteria	
Group differences	
Specific ethnicity (e.g., Indian / Pakistani / White British)	
Total sample size	
Notes	
**Population baseline characteristics**	
**Field**	**Extracted data**
Mean age (years) ± SD	
Sex distribution (% male/female)	
Mean BMI ± SD	
Duration of T2DM	
Medication use (metformin / insulin / not reported)	
HOMA-IR used? (Yes/No)	
HOMA version (HOMA1 / HOMA2 / unclear)	
Mean HOMA-IR ± SD	
HOMA-IR log-transformed? (Yes/No)	
**Outcome data**	
**Field**	**Extracted data**
miRNA name	

Direction of association (Positive / Negative)	
Statistical measure type (Correlation / β coefficient / OR / Group comparison)	
Confidence interval (if reported)	
p-value	
Adjusted or unadjusted	
Covariates adjusted for	
HOMA-IR transformation (log-transformed? Yes/No)	
Correlation coefficient (r or ρ)	
Effect estimate (β or % change)	
Group comparison p-value (for tertile or median comparisons)	
Notes	

patterns.

Supplementary Table S2: Complete miRNA–HOMA-IR Association Data

This table presents complete miRNA–HOMA-IR association data for all 29 associations across 14 included studies, including effect sizes, statistical methods, adjustment status, and methodological notes.

Full circulating miRNA–HOMA-IR association data from included studies (n = 29 associations from 14 studies).

Study (year)	Region / ethnicity	miRNA	Direction	Statistical method	Effect size	p-value	Adjusted?	Notes
Sangalli et al., 2020 [41]	European (Italy)	miR-15a	Positive	Spearman correlation	ρ = 0.30	0.003	Unadjusted	Plasma. No CI. HOMA-IR version unclear; not log-transformed. Cross-sectional only.
Jaeger et al., 2018 [42]	European (Austria/UK)	miR-194	Positive	Spearman correlation	ρ = 0.30	0.0005	Unadjusted	Serum. No CI. HOMA-IR version/log status unclear.
Jaeger et al., 2018 [42]	European (Austria/UK)	miR-192	Positive	Spearman correlation	ρ = 0.29	0.0007	Unadjusted	Serum. No CI. HOMA-IR version/log status unclear.
Willeit et al., 2017 [43]	European (multi-country)	miR-122	Positive	Linear regression (% difference per 1 SD log HOMA-IR)	+67% (95% CI: 39–101)	<0.001	Adjusted (age, sex)	Serum/plasma. Log HOMA-IR (HOMA1). Prospective cohort. Strongest effect estimate in review.
Wang et al., 2014 [44]	European (Sweden)	miR-191	Positive	Pearson correlation	ρ = 0.24	0.04	Unadjusted	Cross-ethnic cohort. Plasma. Log HOMA-IR. No CI. Ethnicity-

								specific data not reported separately.
Wang et al., 2014 [44]	European (Sweden)	miR-320a	Positive	Pearson correlation	ρ = 0.25	0.04	Unadjusted	*As above.
Wang et al., 2014 [44]	European (Sweden)	miR-24	Positive	Pearson correlation	ρ = 0.37	0.002	Unadjusted	*As above.
Wang et al., 2014 [44]	European (Sweden)	miR-29b	Positive	Pearson correlation	ρ = 0.42	0.0003	Unadjusted	Strongest correlation in Wang 2014. Combined-cohort data. Log HOMA-IR. No CI.
Wang et al., 2014 [44]	European (Sweden)	miR-126	Positive	Pearson correlation	ρ = 0.30	0.01	Unadjusted	*As above.
Wang et al., 2014 [44]]	European (Sweden)	miR-150	Positive	Pearson correlation	ρ = 0.24	0.05	Unadjusted	Borderline significance (p = 0.05). Combined-cohort data. Log HOMA-IR. No CI.
Wang et al., 2014 [44]	European (Sweden)	miR-21	Positive	Pearson correlation	ρ = 0.19	0.12 (NS)	Unadjusted	Non-significant. Combined-cohort data. Log HOMA-IR. No CI.
Wang et al., 2014 [44]	European (Sweden)	miR-144	Null	Pearson correlation	ρ = 0.16	0.19 (NS)	Unadjusted	Non-significant. Combined-cohort

								data. Log HOMA-IR. No CI.
Wang et al., 2014 [44]	European (Sweden)	miR-197	Null	Pearson correlation	ρ = 0.16	0.19 (NS)	Unadjusted	Non-significant. Combined-cohort data. Log HOMA-IR. No CI.
Wang et al., 2014 [44]	European (Sweden)	miR-223	Positive	Pearson correlation	ρ = 0.19	0.11 (NS)	Unadjusted	Non-significant. Combined-cohort data. Log HOMA-IR. No CI.
Wang et al., 2014 [44]	European (Sweden)	miR-486-5p	Positive	Pearson correlation	ρ = 0.19	0.13 (NS)	Unadjusted	Non-significant. Combined-cohort data. Log HOMA-IR. No CI.
Wang et al., 2014 [44]	European (Sweden)	miR-15a	Positive	Pearson correlation	ρ = 0.22	0.07 (NS)	Unadjusted	Non-significant. Combined-cohort data. Log HOMA-IR. No CI.
Serik et al., 2023 [45]	European (Ukraine)	miR-126-3p	Negative	Group comparison (HOMA-IR tertiles)	Not reported	0.041	Unadjusted	Lower miR-126-3p in highest HOMA-IR tertile. No coefficient. High ROB.
Šimonienė et al., 2022 [46]	European (Lithuania)	miR-107	Positive	Pearson/Spearman correlation	r = 0.373	0.035	Unadjusted	Serum. HOMA1-style. No CI. No log-transformation stated.
Serik et al., 2021 [47]	European (Ukraine)	miR-221	Negative	Group comparison (HOMA-IR tertiles)	3rd tertile: 0.41 vs	0.028	Unadjusted	Sample type unclear. High ROB. No coefficient or CI.

					1st tertile: 0.70 r.u.			
Serik et al., 2020 [48]	European (Ukraine)	miR-221	Negative	Group comparison (HOMA-IR tertiles)	3rd tertile: 0.41 vs 1st tertile: 0.70 r.u.	0.028	Unadjusted	Same cohort as Serik 2021. Sample type unclear. High ROB. Overlapping study.
Serik et al., 2022 [49]	European (Ukraine)	miR-126-3p	Negative	Spearman correlation + tertile comparison	R = −0.229	r: 0.082 (NS); tertile: 0.041	Unadjusted	Spearman NS; tertile comparison significant. Same cohort as Serik 2023.
Prabu et al., 2020 [50]	South Asian (India)	miR-128	Positive	Pearson correlation	r = 0.183	0.021	Unadjusted	Serum. HOMA1. No CI. Regression models in paper relate to depression score, not HOMA-IR.
Prabu et al., 2015 [51]	South Asian (India)	miR-374a-5p	Positive	Linear regression (β coefficient)	β = 0.1093 (unadj.); β = 0.1082 (sex-adj.)	0.013 (unadj.); 0.015 (sex-adj.)	Unadjusted and sex-adjusted	Serum. HOMA1. Consistent across adjustment. No CI.
Purohit et al., 2021 [52]	South Asian (India)	hsa-miR-330-3p	Positive	Spearman correlation	ρ = 0.498	0.005	Unadjusted	Very small sample (n = 30). Platform/normalisation poorly reported. Overall high ROB.
Sucharita et al., 2019 [53]	South Asian (India)	miR-30d	Positive	Pearson correlation	r = 0.26	0.04	Unadjusted	Plasma. HOMA1-style. No CI. Log-

								transformation status not reported.
Pokharel et al., 2024 [54]	South Asian (Nepal)	miR-192	Positive	Spearman correlation	~0.4 (heatmap only)	<0.01 (heatmap)	Unadjusted	Exact coefficients not reported in publication; author contacted (response pending). Approximate values estimated from heatmap colour scale.
Pokharel et al., 2024 [54]	South Asian (Nepal)	miR-29a	Positive	Spearman correlation	~0.3 (heatmap only)	<0.05 (heatmap)	Unadjusted	As above.
Pokharel et al., 2024 [54]	South Asian (Nepal)	miR-9	Positive (weak)	Spearman correlation	~0.2 (heatmap only)	>0.05 (NS)	Unadjusted	As above. Non-significant.
Pokharel et al., 2024 [54]	South Asian (Nepal)	miR-375	Positive (weak)	Spearman correlation	~0.1 (heatmap only)	>0.05 (NS)	Unadjusted	As above. Non-significant.

Supplementary Table S3. Calculated 95% CIs for the 25 associations plotted in the forest plot

Study (year)	Ethnicity	miRNA	n	Coefficient (type)	Reported *p*	Calculated 95% CI	Significance
Sangalli et al., 2020 [41]	European	miR-15a	76	0.300 (ρ)	0.003	0.080 to 0.492	Sig
Jaeger et al., 2018 [42]	European	miR-192	129	0.290 (ρ)	0.0007	0.123 to 0.441	Sig
Jaeger et al., 2018 [42]	European	miR-194	129	0.300 (ρ)	0.0005	0.134 to 0.450	Sig
Wang et al., 2014 [44]	European	miR-29b	68	0.420 (ρ)	0.0003	0.202 to 0.599	Sig
Wang et al., 2014 [44]	European	miR-24	68	0.370 (ρ)	0.002	0.144 to 0.559	Sig
Wang et al., 2014 [44]	European	miR-126	68	0.300 (ρ)	0.01	0.066 to 0.502	Sig
Wang et al., 2014 [44]	European	miR-320a	68	0.250 (ρ)	0.04	0.012 to 0.461	Sig
Wang et al., 2014 [44]	European	miR-191	68	0.240 (ρ)	0.04	0.002 to 0.453	Sig
Wang et al., 2014 [44]	European	miR-150	68	0.240 (ρ)	0.05	0.002 to 0.453	Sig (borderline)
Wang et al., 2014 [44]	European	miR-15a	68	0.220 (ρ)	0.07	−0.019 to 0.436	NS
Wang et al., 2014 [44]	European	miR-223	68	0.190 (ρ)	0.11	−0.051 to 0.410	NS
Wang et al., 2014 [44]	European	miR-486-5p	68	0.190 (ρ)	0.13	−0.051 to 0.410	NS
Wang et al., 2014 [44]	European	miR-21	68	0.190 (ρ)	0.12	−0.051 to 0.410	NS

Wang et al., 2014 [44]	European	miR-144	68	0.160 (ρ)	0.19	−0.082 to 0.384	NS
Wang et al., 2014 [44]	European	miR-197	68	0.160 (ρ)	0.19	−0.082 to 0.384	NS
Šimonienė et al., 2022 [46]	European	miR-107	108	0.373 (r)	0.035	0.198 to 0.525	Sig
Serik et al., 2021 [47]	European	miR-221	80	−0.360 (r)	0.006	−0.537 to −0.152	Sig
Serik et al., 2022 [49]	European	miR-126-3p	68	−0.229 (r)	0.082	−0.443 to 0.010	NS
Prabu et al., 2020 [50]	South Asian	miR-128	160	0.183 (r)	0.021	0.029 to 0.329	Sig
Purohit et al., 2021 [52]	South Asian	miR-330-3p	30	0.498 (ρ)	0.005	0.168 to 0.728	Sig
Pokharel et al., 2024 [54]	South Asian	miR-192 ‡	46	0.400 (ρ)	<0.01	0.124 to 0.618	Sig
Pokharel et al., 2024 [54]	South Asian	miR-29a ‡	46	0.300 (ρ)	<0.05	0.011 to 0.543	Sig
Sucharita et al., 2019 [53]	South Asian	miR-30d	60	0.260 (r)	0.04	0.007 to 0.482	Sig
Pokharel et al., 2024 [54]	South Asian	miR-9 ‡	46	0.200 (ρ)	>0.05	−0.096 to 0.463	NS
Pokharel et al., 2024 [54]	South Asian	miR-375 ‡	46	0.100 (ρ)	>0.05	−0.196 to 0.379	NS

Note: Pokharel 2024 correlation coefficients were approximated from the published heatmap, exact values were not tabulate by the authors, and no response from the author has been made for this request. CIs derived from these approximations should therefore be interpreted as indicative. Coefficient types (ρ = Spearman's rank correlation; r = Pearson's correlation) were treated equivalently for CIs as all are bounded on [-1,+1] and Fisher's z-transformation is appropriate for each.

Supplementary Table references

41. Sangalli, E.; Tagliabue, E.; La Sala, L.; Prattichizzo, F.; Uccellatore, A.; Spada, D.; Lorino, F.; de Candia, P.; Lupini, S.; Cantone, L.; et al. Circulating microRNA-15a associates with retinal damage in patients with early-stage type 2 diabetes. *Front. Endocrinol.* **2020**, *11*, 254. https://doi.org/10.3389/fendo.2020.00254.
42. Jaeger, A.; Zollinger, L.; Saely, C.H.; Muendlein, A.; Evangelakos, I.; Nasias, D.; Charizopoulou, N.; Schofield, J.D.; Othman, A.; Soran, H.; et al. Circulating microRNAs-192 and -194 are associated with the presence and incidence of diabetes mellitus. *Sci. Rep.* **2018**, *8*, 14274. https://doi.org/10.1038/s41598-018-32274-9.
43. Willeit, P.; Skroblin, P.; Moschen, A.R.; Yin, X.; Kaudewitz, D.; Zampetaki, A.; Barwari, T.; Whitehead, M.; Ramírez, C.M.; Goedeke, L.; et al. Circulating microRNA-122 is associated with the risk of new-onset metabolic syndrome and type 2 diabetes. *Diabetes* **2017**, *66*, 347–357. https://doi.org/10.2337/db16-0731.
44. Wang, X.; Sundquist, J.; Zöller, B.; Memon, A.A.; Palmér, K.; Sundquist, K.; Bennet, L. Determination of 14 circulating microRNAs in Swedes and Iraqis with and without diabetes mellitus type 2. *PLoS ONE* **2014**, *9*, e86792. https://doi.org/10.1371/journal.pone.0086792.
45. Serik, S.A.; Mavrycheva, N.; Riabukha, V.; Malko, V.; Bondar, T.; Chenchik, T. The association of circulating microRNA-126 with inflammation and insulin resistance in patients with coronary artery disease with type 2 diabetes mellitus. *Atherosclerosis* **2023**, *379*, S139. https://doi.org/10.1016/j.atherosclerosis.2023.06.479.
46. Šimonienė, D.; Stukas, D.; Daukša, A.; Veličkienė, D. Clinical role of serum miR-107 in type 2 diabetes and related risk factors. *Biomolecules* **2022**, *12*, 558. https://doi.org/10.3390/biom12040558.
47. Serik, S.A.; Serdobinska-Kanivets, E.; Kurinna, O.; Bondar, T. Circulating microRNA-27a, -126, -146a, -221 levels in coronary artery disease and type 2 diabetes mellitus. *Metabolism* **2021**, *116*, 154544. https://doi.org/10.1016/j.metabol.2020.154544.
48. Serik, S.A.; Serdobinska-Kanivets, E.M.; Kurinna, O.H.; Tkachenko, O.V. Circulating microRNA-27a, -221 in coronary artery disease and type 2 diabetes mellitus. *Am. Heart J.* **2020**, *229*, 173–174. https://doi.org/10.1016/j.ahj.2020.10.040.
49. Serik, S.A.; Mavrycheva, N.R.; Bondar, T.M. Circulating microRNA-126 in patients with ischemic heart disease with type 2 diabetes mellitus and its relationship with glucometabolic disorders. *Zaporozhye Med. J.* **2022**, *24*, 501–508. https://doi.org/10.14739/2310-1210.2022.5.257413.
50. Prabu, P.; Poongothai, S.; Shanthirani, C.S.; Anjana, R.M.; Mohan, V.; Balasubramanyam, M. Altered circulatory levels of miR-128, BDNF, cortisol and shortened telomeres in patients with type 2 diabetes and depression. *Acta Diabetol.* **2020**, *57*, 799–807. https://doi.org/10.1007/s00592-020-01486-9.
51. Prabu, P.; Rome, S.; Sathishkumar, C.; Aravind, S.; Mahalingam, B.; Shanthirani, C.S.; Gastebois, C.; Villard, A.; Mohan, V.; Balasubramanyam, M. Circulating miRNAs of "Asian Indian phenotype" identified in subjects with impaired glucose tolerance and patients with type 2 diabetes. *PLoS ONE* **2015**, *10*, e0128372. https://doi.org/10.1371/journal.pone.0128372.
52. Purohit, P.; Roy, D.; Modi, A.; Choudhary, R.; Shukla, R.P.; Sharma, P. Circulating and visceral adipose tissue miR-330-3p is associated with insulin resistance in type II diabetes mellitus. *Metabolism* **2021**, *116*, 154552. https://doi.org/10.1016/j.metabol.2020.154552.
53. Sucharita, S.; Ashwini, V.; Prabhu, J.S.; Avadhany, S.T.; Ayyar, V.; Bantwal, G. The role of circulating microRNA in the regulation of beta cell function and insulin resistance among Indians with type 2 diabetes. *Indian J. Endocrinol. Metab.* **2019**, *22*, 770–773. https://doi.org/10.4103/ijem.IJEM_162_18.
54. Pokharel, D.R.; Maskey, A.; Kafle, R.; Batajoo, A.; Dahal, P.; Raut, R.; Adhikari, S.; Manandhar, B.; Das Manandhar, K. Evaluation of circulating plasma miR-9, miR-29a, miR-192, and miR-375 as potential biomarkers for predicting prediabetes and type 2 diabetes in Nepali adult population. *Noncoding RNA Res.* **2024**, *9*, 1324–1332. https://doi.org/10.1016/j.ncrna.2024.07.001.
